# Red blood cell transfusion practice and cardiac comorbidities in patients with myelodysplastic syndromes

**DOI:** 10.1111/trf.18391

**Published:** 2025-08-29

**Authors:** Allison Mo, Erica M. Wood, Jake Shortt, Zoe K. McQuilten

**Affiliations:** ^1^ Transfusion Research Unit, School of Public Health & Preventive Medicine, Faculty of Medicine, Nursing & Health Sciences Monash University Melbourne Australia; ^2^ Monash Hematology, Monash Health Melbourne Australia; ^3^ Austin Pathology and Department of Haematology, Austin Health Melbourne Australia; ^4^ Department of Medicine, School of Clinical Sciences, Faculty of Medicine, Nursing & Health Sciences Monash University Melbourne Australia; ^5^ Department of Haematology Alfred Health Melbourne Australia

**Keywords:** RBC transfusion, transfusion practices (adult), transfusion practices (oncology‐hematology)

## Abstract

**Background:**

Evidence guiding optimal transfusion practice for patients with myelodysplastic syndromes (MDS) is lacking. Many patients have concurrent cardiac disease. Data on practice are sparse. We aimed to describe the use of red blood cell (RBC) transfusion and the prevalence of cardiac comorbidities in patients with MDS within Australia's largest public hospital network to better understand real‐world practices and outcomes.

**Methods:**

We conducted a retrospective cohort study of patients aged ≥18 years with MDS, MDS/myeloproliferative overlap neoplasm or chronic myelomonocytic leukemia admitted from 2016 to 2018 to determine RBC transfusion‐related endpoints.

**Results:**

One hundred and seventy‐nine patients (median age 78 years, 61.5% male) were included, with a median follow‐up of 46 weeks. Of these, 102 (57.0%) received RBC transfusion. Transfused patients had lower presenting Hb (87 vs. 105 g/L, *p* < 0.0001), higher rates of cardiac disease (29.4% vs. 12.9%, *p* = 0.009) and 5‐azacytidine use (31.4% vs. 13.0%, *p* = 0.004). Sixty‐five patients (36.3%) received outpatient RBC transfusions, with a median of 2 units RBC per transfusion and 14 days between transfusions. The median pre‐transfusion Hb was 80 g/L (IQR 74–86 g/L). Forty patients (22.4%) had evidence of cardiac disease, with similar pre‐transfusion Hb for patients with and without cardiac disease (median Hb 79 g/L vs. 81 g/L, *p* = 0.1).

**Discussion:**

Patients with MDS frequently require RBC transfusion, and restrictive transfusion strategies predominate despite many patients having cardiac comorbidities. Further research is needed to address optimal transfusion strategies in such patients and associated cardiac outcomes.

## 1 INTRODUCTION

Myelodysplastic syndromes (MDS) are hematological malignancies characterized by cytopenias and bone marrow failure, usually affecting the elderly. Management is largely supportive, as the only curative treatment, bone marrow transplantation (BMT), is unsuitable for many due to age and comorbidities. Anemia is common and associated with worse quality of life (QoL).^1^ Red blood cell (RBC) transfusion aims to improve patient symptoms. Although newer therapies such as erythropoiesis‐stimulating agents (ESAs) and erythroid maturation agents (EMAs)^2^ can improve MDS‐related anemia, these may not be available or suitable for all patients and are costly; thus, transfusion remains vital to management.

Chronic RBC transfusions may have complications, such as transfusion reactions, alloimmunization, and iron overload, and can be costly.^3^ Additionally, patients may find repeated blood tests and transfusions burdensome,^4^ contributing to the time toxicity of cancer treatments.^5^

The evidence base guiding RBC transfusion in MDS is sparse. Cochrane reviews of RBC transfusion hemoglobin (Hb) thresholds in different clinical settings^6^ and in patients with hematological malignancy treated with chemotherapy/radiotherapy^7^ were unable to make definitive conclusions for MDS patients. Although transfusion is given to improve QoL, systematic reviews have found no definitive evidence that a higher Hb transfusion strategy improves QoL,^8^, ^9^ though significant limitations were identified in included studies. This uncertainty is reflected in national^10^ and international guidelines,^11^ which are unable to recommend specific transfusion thresholds for chronically transfused outpatients, whereas restrictive strategies (Hb threshold <70–80 g/L) are recommended in acute settings.^10^, ^11^ Additionally, cardiac comorbidities are common in elderly patients but optimal transfusion strategies to reduce cardiac complications are unknown.^6^, ^12^

Despite the paucity of clear guidelines and data, in our 2017 Australasian MDS clinical practice survey, clinicians reported they would typically transfuse patients at Hb thresholds of <80 g/L^13^, consistent with a restrictive strategy. An international survey showed variability in Hb thresholds, with the commonest being <80 g/L.^4^ As these surveys are self‐reported, with the potential for responder bias or inaccuracies, we aimed to conduct a study of real‐world transfusion practice, to better understand practices, transfusion thresholds, transfusion burden, and cardiac comorbidity prevalence in patients with MDS.

## 2 METHODS

### 2.1 Study design and patient population

Following ethics approval (HREC reference RES‐17‐0000‐778Q), we conducted a retrospective cohort study of all patients aged ≥18 years with a diagnosis of MDS, chronic myelomonocytic leukemia (CMML), and other MDS/myeloproliferative overlap neoplasms (MDS/MPN) per World Health Organization (WHO) criteria^14^ admitted to the Monash Health hospital network in Melbourne, Australia, from August 1, 2016 to July 31, 2018. This time period was selected to coincide with the timing of our Australasian practice survey.^13^ Monash Health is a tertiary‐level public hospital network and includes five adult hospitals, serving approximately 25% of Melbourne's population, with >4 million patient annual admission episodes.

Patients were identified through hospital records. In Australia, all hospital diagnoses and procedures are recorded using the International Statistical Classification of Diseases and Related Health Problems, Tenth Revision (ICD‐10‐AM).^15^ We retrieved all admission records from Monash Health during the study period with ICD‐10‐AM codes for MDS, CMML, and MDS/MPN overlap (Table S1). The diagnosis was confirmed via patient medical records.

### 2.2 Data collection

All hospital admission episodes following the first diagnosis of MDS/CMML in our institution's records were extracted, including demographics, diagnoses, and RBC transfusion code (ICD‐10‐AM #1370602). This included both multi‐day “inpatient” hospitalizations and single‐day “outpatient” admissions in the hospital day unit.

Hb on the first day of hospital admission was recorded, for all included episodes.

ESAs, 5‐azacytidine (AZA; the only hypomethylating agent which was reimbursed under the Australian Pharmaceutical Benefits Scheme [PBS] during the time of the study), lenalidomide (PBS‐reimbursed for patients with deletion 5q MDS) and iron chelation prescriptions were obtained from pharmacy records. ESAs are not reimbursed under the PBS schedule for MDS in Australia. EMAs were not available during the study period outside a clinical trial.

### 2.3 Anemia management

Transfusions were administered at the discretion of treating clinicians. Hospital policies reference national guidelines, which do not specify a particular Hb threshold in this setting (due to lack of evidence).^10^ All RBC units issued by the national blood service (Australian Red Cross Lifeblood) are leucodepleted.

### 2.4 Definition of inpatient versus outpatient transfusion episodes

RBC transfusion may occur during an “outpatient” day unit episode, or as part of a multi‐day “inpatient” hospitalization admission. We particularly focused on outpatient episodes as these transfusions are typically planned in stable outpatients, whereas intercurrent illnesses (e.g., infection or bleeding) are more likely to affect transfusions for inpatients.

### 2.5 Definition of cardiac events and transfusion reactions

Cardiac events and transfusion reactions were identified by ICD‐10‐AM codes (Table S1). Ischemic cardiac disease included any diagnostic codes containing the terms myocardial infarction, angina, ischemic heart disease or coronary thrombosis. Cardiac failure diagnostic codes included the terms pulmonary edema, heart failure, ventricular failure, fluid overload, cardiogenic, shock or generalized/localized edema. This approach using ICD‐10‐AM codes only identifies events which require hospitalization, or occurring during hospitalization.

### 2.6 Statistical analysis

Statistical analyses were performed using Stata version 11.0 (Statacorp LP, College Station, Tex). Patients were censored at BMT or acute myeloid leukemia (AML) transformation (≥20% blasts), as these events are likely to affect transfusion requirements. Comparisons for significance of differences were performed using Chi‐square or Mann–Whitney tests, and *p* < 0.05 was considered statistically significant.

## 3 RESULTS

**FIGURE 1:**
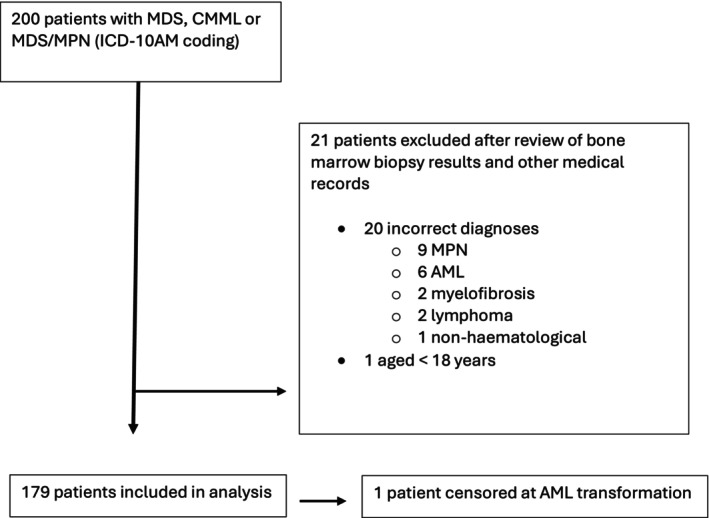
Consort diagram of study patient selection.

From August 1, 2016 to July 31, 2018, 200 patients were identified. After excluding 21 patients (20 for non‐MDS diagnoses and one patient aged <18 years; Figure 1), 179 patients with a total of 804 admission episodes were included. Fifty‐five patients died (30.7%). Nine patients (5.0%) were lost to follow‐up (defined as no contact with the health service for >12 months). Median follow‐up time was 32 weeks (IQR 12–55 weeks).

### 3.1 Patient characteristics

Table 1 shows clinical characteristics. Median age was 78 years (IQR 71–84); 110 (61.5%) were male. Forty patients (22.3%) had cardiac disease documented during follow‐up.

**TABLE 1:** Clinical characteristics.

Characteristic	Entire cohort *N* = 179	Received RBC transfusion *N* = 102	No RBC transfusion *N* = 77
Age in years, median (IQR)	78 (71–84)	78 (73–84)	78 (69–84)
Male *n* (%)	110 (61.5%)	67 (65.7%)	43 (55.8%)
Female *n* (%)	69 (38.5%)	35 (34.3%)	34 (44.2%)
Cardiac disease at any time during follow‐up, *n* (%)	40 (22.3%)	30 (29.4%)	10 (12.9%)
Ischaemic heart disease^a^	31 (17.3%)	25 (24.5%)	6 (7.8%)
Cardiac failure^a^	36 (20.1%)	26 (25.5%)	10 (13.0%)
MDS classification, *n* (%)			
MDS‐SLD	1 (0.6%)	1 (1.0%)	0
MDS‐RS‐SLD	5 (2.8%)	2 (2.0%)	3 (3.9%)
MDS‐RS‐MLD	13 (7.3%)	6 (5.9%)	7 (9.1%)
MDS‐MLD	40 (22.3%)	22 (21.6%)	18 (23.4%)
MDS‐EB1	7 (3.9%)	5 (4.9%)	2 (2.6%)
MDS‐EB2	19 (10.6%)	13 (12.8%)	6 (7.8%)
del‐5q	1 (0.6%)	1 (1.0%)	0
CMML‐0	8 (4.5%)	2 (2.0%)	6 (7.8%)
CMML‐1	11 (6.2%)	5 (4.9%)	6 (7.8%)
CMML‐2	9 (5.0%)	3 (2.9%)	6 (7.8%)
Other MDS/MPN overlap	12 (6.7%)	10 (9.8%)	2 (2.6%)
Therapy‐related MDS	5 (2.8%)	3 (2.9%)	2 (2.6%)
Unknown (bone marrow biopsy not available)	48 (26.8%)	29 (28.4%)	19 (24.7%)
IPSS‐R category, *n* (%)^b^			
Very low risk	21 (11.7%)	7 (6.9%)	14 (18.2%)
Low	50 (27.9%)	28 (27.5%)	22 (28.6%)
Intermediate	15 (8.4%)	10 (9.8%)	5 (6.5%)
High	9 (5.0%)	6 (5.9%)	3 (3.9%)
Very high	9 (5.0%)	7 (6.9%)	2 (2.6%)
Unknown	75 (41.9%)	44 (43.1%)	31 (40.0%)
Hemoglobin (g/L) at first admission, median (IQR)	93 (83–108)	87 (77–95)	105 (95–117)
ESA, *n* (%)	11 (6.2%)	9 (8.8%)	2 (2.6%)
Iron chelation, *n* (%)	12 (6.7%)	10 (9.8%)	2 (2.6%)
Azacitidine, *n* (%)	42 (23.5%)	32 (31.4%)	10 (13.0%)
Lenalidomide, *n* (%)	1 (0.56%)	1 (0.98%)	0
Number of outpatient day unit episodes, median (IQR)	1 (0–3)	2 (0–4)	1 (0–1)
Number of inpatient hospital admissions, median (IQR)	1 (0–2)	1 (0–3)	0 (0–1)

^a^ 27 patients had both ischemic cardiac disease and cardiac failure.

^b^ IPSS‐R category unknown due to failed cytogenetics or the diagnostic bone marrow biopsy report not being available.

Abbreviations: AZA, 5‐azacytidine; CMML, chronic myelomonocytic leukemia; Del‐5q, MDS with isolated del(5q); ESA, erythropoiesis stimulating agents; IPSS‐R Revised International Prognostic Scoring System (IPSS‐R); IQR, interquartile range; MDS‐EB, MDS with excess blasts; MDS‐MLD, MDS with multilineage dysplasia; MDS/MPN overlap, MDS/myeloproliferative neoplasm overlap; MDS‐SLD, MDS with single lineage dysplasia; MDS‐RS‐MLD, MDS with ring sideroblasts and multilineage dysplasia; MDS‐RS‐SLD, MDS with ring sideroblasts and single lineage dysplasia.

The commonest MDS subtype was MDS with multilineage dysplasia (*n* = 40, 22.3%). Forty‐eight patients (26.8%) had unknown classification due to unavailable bone marrow biopsy results (e.g., performed elsewhere). R‐IPSS risk categories^16^ were high to very high in 18 (10%) (Table 1).

The median Hb at first admission was 93 g/L (IQR 83‐108 g/L). Eleven patients (6.2%) received ESA, 12 patients (6.7%) received iron chelation therapy, and 42 (23.5%) received AZA during the study period. One patient (with del5q MDS) received lenalidomide. No patients received both ESA and AZA or lenalidomide.

One hundred and two patients (57.0%) received one or more RBC transfusions during the study period, during an inpatient admission or outpatient episode. Transfused patients had a median Hb of 87 g/L at first admission, compared to Hb 105 g/L for those who were not transfused (*p* < 0.0001). Of the 102 transfused patients, 21 (20.6%) became transfusion independent, defined as not requiring RBC transfusion for a minimum of 8 weeks^17^ maintained until the end of follow‐up.

### 3.2 Patients receiving outpatient RBC transfusion

Sixty‐five patients (36.3%) received outpatient RBC transfusion over 388 episodes, using a total of 717 RBC units. Table 2 shows outpatient transfusion characteristics. Twenty‐three patients (35.4%) received AZA and six (9.2%) received ESA; no patients received both. The median number of outpatient transfusion episodes per patient was three (IQR 1–7), with a median of two RBC units transfused per episode and 14 days between transfusions. The median pre‐transfusion Hb was 80 g/L (IQR 74–86 g/L), with a median 24‐h post‐transfusion Hb increment of +13 g/L.

**TABLE 2:** Characteristics of patients receiving outpatient RBC transfusions.

No of patients	65
Age, years, median (IQR)	77 (72–82)
Sex, male, *n* (%)	41 (63.1%)
Azacitidine use, *n* (%)	23 (35.4%)
ESA use, *n* (%)	6 (9.2%)
Total no. of outpatient RBC transfusion admissions	388
No. of outpatient RBC transfusion admissions per patient, median (IQR; range)	3 (IQR 1–7; range 1–55)
No. of RBC units transfused (total)	717
No. RBC units transfused per episode, median (IQR; range)	2 (IQR 1–2; range 1–4)
Interval (days) between transfusion episodes, median (IQR; range)	14 (IQR 7–23; range 2–314)
Pre‐transfusion Hb (g/L)^a^, median (IQR; range)	80 (IQR 74–86; range 28–105)
24‐h post‐transfusion Hb increment (g/L), median (IQR; range)^b^	+13 (IQR +2 to +21; range −2 to +48)

^a^ Recorded in 370 transfusion episodes.

^b^ Recorded in 17 patients. It is not routine practice to measure a 24‐h Hb increment at our hospitals; however, patients may have repeat Hb testing if they subsequently present to the hospital or Emergency Department, have routine outpatient pathology done, or were specifically called back for a repeat Hb.

### 3.3 Transfusion reactions

No transfusion reactions were recorded in the coding data during the study period.

### 3.4 Cardiac comorbidities prevalence and transfusion thresholds

Forty patients (22.3%) had cardiac disease at any time during the study period, either documented as a comorbid condition or a new diagnosis. There was no difference in pre‐transfusion Hb for patients with versus without any cardiac disease (median Hb 79 g/L vs. 81 g/L, *p* = 0.1), or when subdivided into those with ischemic cardiac disease (median Hb 81 g/L vs. 80 g/L, *p* = 0.7) or cardiac failure (81 g/L vs. 70 g/L, *p* = 0.12).

## 4 DISCUSSION

This study shows that patients with MDS frequently require RBC transfusion, and a restrictive transfusion threshold is common, with a median pre‐transfusion Hb of 80 g/L, in keeping with our^13^ and other surveys.^4^ Additionally, cardiac comorbidities are common, seen in nearly one‐quarter of patients.

Despite the high transfusion burden, with over one‐third of patients with MDS receiving outpatient RBC transfusion, there are limited clinical trial data on optimal transfusion strategies. Although not powered for clinical outcomes, the combined results of two pilot studies (*n* = 66) showed liberal transfusion thresholds improved QoL in selected domains, compared to restrictive thresholds.^18^, ^19^, ^20^ Another MDS transfusion threshold study was terminated early due to slow recruitment.^21^ One ongoing study is investigating whether maintaining a more stable Hb (rather than a specific threshold) with weekly transfusion also impacts patient outcomes.^22^

The high prevalence of cardiac comorbidities in patients with MDS, and potential impacts on transfusion outcomes, is also important. Chronic anemia in MDS may lead to cardiac dysfunction and remodeling due to pathophysiological adaptions.^23^ Cardiac iron overload also occurs in transfusion‐dependent MDS (17% in one study).^24^ There is the risk of transfusion‐associated circulatory overload (TACO) with each transfusion. However, optimal transfusion practice in patients with cardiac comorbidities remains uncertain.^6^ In the recent Myocardial Ischemia and Transfusion (MINT) trial in patients with acute MI and anemia, although a liberal transfusion strategy did not reduce the risk of recurrent MI or death at 30 days, it was consistently favored over a restrictive strategy in point estimates for death, cardiac death, recurrent MI, and hospital readmission, without safety concerns.^25^ Guidelines highlight ongoing uncertainty regarding the safety of restrictive thresholds in patients with ischemic cardiac disease.^11^, ^26^

No transfusion reactions were documented in our study, based on ICD‐10AM codes. This may be due to under‐recognition, under‐reporting, and/or reflect ICD‐10AM limitations as there are only five relevant codes (ABO incompatibility, Rh incompatibility, anaphylactic shock due to serum, other serum reaction, shock during or result from a procedure not otherwise specified; Table S1).

Limitations of our study include the retrospective design, the possibility of missing/incorrect diagnostic codes (e.g., incorrect code recorded for transfusion), lack of QoL data, and we could not capture complications occurring outside the hospital setting, though these are likely less severe. Additionally, ESAs and EMAs are not currently reimbursed in Australia for patients with MDS; hence, our findings may not be generalizable internationally. However, even in countries in which ESAs are widely available, transfusion rates in patients with MDS remain high.^4^

Strengths of the study include real‐world data which follows up on our clinician survey findings, and detailed laboratory data regarding transfusion thresholds and transfusion rates in MDS outpatients.

## 5 CONCLUSIONS

Patients with MDS are typically elderly, frequently have cardiac comorbidities, and are chronically anemic. Despite the lack of evidence supporting any particular transfusion strategy, restrictive transfusion strategies are commonly used, including in patients with cardiac disease. Given our findings and emerging evidence suggesting liberal transfusion may be preferred in patients with subtypes of cardiac disease, there is a need for research to investigate the optimal transfusion strategy in patients with MDS, particularly focusing on cardiac outcomes.

## AUTHOR CONTRIBUTIONS

Allison Mo, Zoe K. McQuilten, Erica M. Wood, and Jake Shortt developed the study concept and design. Allison Mo performed the data collection. Allison Mo and Zoe K. McQuilten conducted the data analysis. All authors wrote and edited the manuscript.

## FUNDING INFORMATION

Allison Mo receives PhD scholarship funding from the National Health and Medical Research Council (NHMRC), National Blood Authority, Monash University, and Haematology Society of Australia and New Zealand. Jake Shortt and Zoe K. McQuilten are supported by Australian NHMRC Emerging Leadership Fellowships (grant no. Zoe K. McQuilten: 1194811, Jake Shortt: 2009177). Erica M. Wood is supported by an NHMRC Leadership Fellowship (grant no. 1177784). Monash Health acknowledges the support of a donation from Dr. Alexander Baxter for its MDS research program.

## CONFLICT OF INTEREST STATEMENT

Allison Mo does not have any conflict of interest to disclose for the submitted work. Jake Shortt has served on advisory boards for Pfizer, Bristol Myers Squibb, Otsuka, Mundipharma, and Novartis. Jake Shortt has received research funding from Astex Pharmaceuticals. Jake Shortt has received speaker's fees from Mundipharma and Novartis (all disclosures for Jake Shortt are outside of the submitted work). Erica M. Wood and Zoe K. McQuilten have received research funding to their institution from: Abbvie, Amgen, Antengene, AstraZeneca, Beigene, Bristol‐Myers Squibb, CSL Behring, Gilead, GSK, Janssen‐Cilag, Novartis, Pfizer, Roche, Sanofi, and Takeda, and research support from Sobi, all outside the submitted work.

## Supporting information


**TABLE S1.** ICD‐10‐AM diagnostic codes.
