# Supplementary material for: Red blood cell transfusion practice and cardiac comorbidities in patients with myelodysplastic syndromes
Source: Transfusion. 2025 Aug 29;65(10):1803–9. doi: 10.1111/trf.18391 (PMC12531908; doi:10.1111/trf.18391)
Supplement: Supplementary file 1 — TABLE S1. ICD‐10‐AM diagnostic codes. [file TRF-65-1803-s001.docx]

**Supplementary table 1: ICD-10-AM diagnostic codes**

| **ICD-10AM CODE** | **DIAGNOSIS** |
| --- | --- |
| **MDS, CMML, MDS/MPN** | |
| C931 | Chronic myelomonocytic leukaemia |
| C9310 | Chronic myelomonocytic leukaemia not having achieved remission |
| C9311 | Chronic myelomonocytic leukaemia, in remission |
| C9312 | Chronic myelomonocytic leukaemia, in relapse |
| D46 | Myelodysplastic syndromes |
| D460 | Refractory anaemia without ring sideroblasts, so stated |
| D461 | Refractory anaemia with ring Sideroblasts |
| D462 | Refractory anaemia with excess of blasts [RAEB] |
| D46 | Refractory anaemia with excess of blasts [RAEB] 1 |
| D464 | Refractory anaemia, unspecified |
| D465 | Refractory anaemia with multi-lineage dysplasia |
| D466 | Myelodysplastic syndrome with isolated del(5q) chromosomal abnormality |
| D467 | Other myelodysplastic syndromes |
| D469 | Myelodysplastic syndrome, unspecified |
| C946 | Myelodysplastic and myeloproliferative disease, not elsewhere classified |
| C9460 | Myelodysplastic and myeloproliferative disease, not elsewhere classified, without mention of remission |
| C9461 | Myelodysplastic and myeloproliferative disease, not elsewhere classified, in remission |
| **TRANSFUSION REACTIONS** | |
| T803 | ABO incompatibility reaction |
| T804 | Rh incompatibility reaction |
| T805 | Anaphylactic shock due to serum |
| T806 | Other serum reactions |
| T811 | Shock during or resulting from a procedure, not elsewhere classified |
| **ISCHEMIC HEART DISEASE** | |
| I20 | Angina pectoris |
| I200 | Unstable angina |
| I201 | Angina pectoris with documented spasm |
| I208 | Other forms of angina pectoris |
| I209 | Angina pectoris unspecified |
| I21 | Acute myocardial infarction |
| I210 | Acute transmural myocardial infarction of anterior wall |
| I211 | Acute transmural myocardial infarction of inferior wall |
| I212 | Acute transmural myocardial infarction of other sites |
| I213 | Acute transmural MI of unspecified site |
| I214 | Acute subendocardial myocardial infarction |
| I219 | Acute myocardial infarction unspecified |
| I22 | Subsequent myocardial infarction |
| I220 | Subsequent myocardial infarction of anterior wall |
| I221 | Subsequent myocardial infarction of inferior wall |
| I228 | Subsequent myocardial infraction of other sites |
| I229 | Subsequent myocardial infarction of unspecified site |
| I24 | Other acute ischaemic heart disease |
| I240 | Coronary thrombosis not resulting in myocardial infarction |
| I248 | Other forms of acute ischaemic heart disease |
| I249 | Acute ischaemic heart disease unspecified |
| I252 | Old myocardial infarction |
| **CARDIAC FAILURE** | |
| E877 | Fluid overload |
| I50 | Heart failure |
| I500 | Congestive heart failure |
| I501 | Left ventricular failure |
| I509 | Heart failure unspecified |
| J80 | Adult respiratory distress syndrome |
| J81 | Pulmonary oedema |
| R570 | Cardiogenic shock |
| R600 | Localised oedema |
| R601 | Generalised oedema |
